# Genotypic and Allelic Frequencies of Hereditary Cataract in the Italian Population of Australian Shepherd and Miniature American Shepherd Dogs

**DOI:** 10.3390/ani15121778

**Published:** 2025-06-17

**Authors:** Maria Grazia De Iorio, Giulietta Minozzi, Sara Ghilardi, Stefano Frattini, Mara Bagardi, Paola Giuseppina Brambilla, Alessandra Paganelli, Maria Cristina Cozzi, Francesca Vecchi, Michele Polli

**Affiliations:** 1 Department of Veterinary Medicine and Animal Sciences, University of Milan, 26900 Lodi, Italy; maria.deiorio@unimi.it (M.G.D.I.); sara.ghilardi@unimi.it (S.G.); mara.bagardi@unimi.it (M.B.); paola.brambilla@unimi.it (P.G.B.); cristina.cozzi@unimi.it (M.C.C.); francesca.vecchi2002@gmail.com (F.V.); michele.polli@unimi.it (M.P.); 2 Vetogene Laboratory—ENCI Servizi SRL, 20139 Milan, Italy; sfrattini.vetogene@enciservizi.it (S.F.); apaganelli.vetogene@enciservizi.it (A.P.)

**Keywords:** hereditary cataract, Australian Shepherd, Miniature American Shepherd, genetics, allelic frequency

## Abstract

Hereditary cataract is a progressive ocular disorder that is present also in Australian Shepherd and Miniature American Shepherd dogs, primarily caused by a mutation in the *HSF4* gene. This study analyzed 233 Australian Shepherd dogs tested in Italy between 2020 and 2024 to evaluate genotypic and allelic frequencies of the main causative mutation. DNA samples were collected and tested, classifying individuals as homozygous wild-type, heterozygous, or homozygous mutant. The overall mutant allele frequency was 6.01%. Furthermore, a small subset of 13 Miniature American Shepherds was analyzed and genetic tests revealed that they were all homozygous wild type, suggesting no presence of the causative allele in this small sample. These findings confirm the importance of genetic testing in dog populations emphasizing the need for responsible selection practices to further minimize the disease’s impact.

## Simple Summary

Hereditary cataract is an eye disease that can cause vision problems in dogs, including Australian Shepherds and Miniature American Shepherds. This condition is linked to changes in the *HFS4* gene. This condition is associated with a specific mutation in the *HSF4* gene. In this study, 233 Australian Shepherds in Italy were genetically tested to determine how frequently this mutation appears in the population The results showed that a small proportion of the dogs carried the mutation, which means they could pass it on to their offspring. Additionally, 13 Miniature American Shepherds were tested, and none were found to carry the mutation. These findings provide useful information about the distribution of this genetic variant and highlight the value of genetic screening. Identifying carriers can support responsible breeding practices, helping to reduce the incidence of hereditary cataract and improve the overall health and quality of life of future dog populations.

## 1. Introduction

The Australian Shepherd dog breed, despite its name, originated in the United States (ASCA). Its intelligence, versatility, and strong working abilities have contributed significantly to its increasing popularity worldwide [1]. In Italy, the breed has seen remarkable increase in registered dogs, with approximately 4003 individuals registered in 2023 [2]. Although generally considered healthy, Australian Shepherds are predisposed to several hereditary diseases, among which hereditary cataract is one of the most prevalent [3].

Hereditary cataract (HC) is a genetic disorder affecting numerous dog breeds, characterized by progressive lens opacification, which can lead to severe visual impairment or blindness [4,5]. Notably, the age of onset, rate of progression, and anatomical location of cataracts can vary significantly between breeds and even within breeds [6]. In the Australian Shepherds, HC typically presents as bilaterally symmetrical opacities primarily located in the posterior cortex of the lens, although individual variability in clinical manifestation remains substantial [7].

The main genetic factor responsible for HC in dogs is the heat shock transcription factor 4 (*HSF4*) gene, located on chromosome 5 (NC_051809.1: 82,635,528–82,626,129) [8]. In many dog breeds, such as the Staffordshire Bull Terriers, Boston Terriers, and French Bulldogs, HC results from a single nucleotide insertion in exon 9 of the *HSF4* gene, which follows an autosomal recessive inheritance pattern [9,10]. Conversely, in Australian Shepherds, Miniature American Shepherds, and Toy Australian Shepherds, HC is associated with a different mutation, specifically, a single-base deletion in the same region of the *HSF4* gene (c.971delC), located at position g.85286582, and displays an autosomal dominant inheritance pattern with incomplete penetrance, meaning that not all carriers will develop clinical signs [11,12,13]. Studies in humans have similarly indicated that mutations occurring within the DNA-binding domain of the *HSF4* gene are generally linked to autosomal dominant congenital or juvenile cataracts, whereas mutations located in other regions of the gene tend to result in autosomal recessive cataracts with earlier onset [14,15,16].

Despite the differences in inheritance patterns, both types of mutations lead to the formation of a premature stop codon and consequently produce a truncated, nonfunctional protein [9,10,17]. However, in other breeds, such as the Havanese, cataract appears to be associated with different genetic loci, specifically CFA20 and CFA21 [18]. Additionally, recent findings in Australian Shepherds have identified another potential genetic contributor, a single nucleotide polymorphism (SNP) located in the intron 5 of the *SCFD2* gene on chromosome 13, which has also been associated with HC. Nevertheless, the *HSF4* mutation remains the primary genetic determinant [19].

Given the non-congenital nature of HC in Australian Shepherds, genetic testing plays a crucial role in identifying carriers and preventing the increase in this mutation through selective breeding practices. The current study aimed to evaluate the prevalence of the *HSF4* mutation responsible for hereditary cataracts in Australian Shepherds and Miniature American Shepherds (a breed derived from smaller Australian Shepherds) on dogs tested in Italy from 2020 to 2024. Furthermore, the study investigated differences in the prevalence of the *HSF4* mutation based on breed, sex, coat color, age, and year of testing. The findings will contribute to a deeper understanding of the *HSF4* mutation frequency and its implications in breeding strategies.

## 2. Materials and Methods

### 2.1. Sampling

This study is a retrospective observational analysis based on data collected between 2020 and 2024. It includes DNA test results for the *HSF4* gene mutation, obtained from Australian Shepherds and Miniature American Shepherds across Italy during this period. A total of 246 dogs were analyzed, comprising 233 Australian Shepherds and 13 Miniature American Shepherds.

DNA testing was requested by owners, breeders, or veterinarians to determine the genetic status of the dogs. Most of the subjects in this study belonged to private owners who sought genetic testing for breeding purposes or to obtain genetic insights before a potential clinical diagnosis. All DNA tests were performed at Vetogene Laboratory, a commercial laboratory that serves as an official reference center for DNA testing for the Italian Kennel Club (Ente Nazionale Cinofilia Italiana, ENCI) and acts as the reference laboratory for the University of Milan.

Samples were collected by veterinarians using EDTA blood tubes or Vetkard cards for blood samples, and GenoTube^®^ buccal swabs for saliva collection (Thermo Fisher, Waltham, MA, USA). In addition to genetic data, information on sex, age, coat color, and breed was recorded. However, due to the significant imbalance in sample size between the two breeds (233 vs. 13 dogs), further statistical analyses were conducted on the Australian Shepherds and Miniature American Shepherds as separate groups.

No ethical approval was required, as the data were obtained from general clinical practice and provided by the laboratory. Written consent was obtained from owners, authorizing the use of the genetic information for research purposes.

### 2.2. DNA Genotyping

DNA was extracted at Vetogene Laboratory, using the E.Z.N.A.^®^ Blood DNA Purification Kit (Omega Bio-tek, Norcross, GA, USA), following standard protocols. The extracted DNA were subsequently sent to EuroVetGene Molecular Diagnostics, an accredited commercial laboratory, for Real-Time PCR testing to identify the genotype based on the melting curve analysis of the *HSF4* gene. The genotyping was further validated through direct sequencing using the Sanger method to confirm the presence of the deletion.

Based on the results, samples were classified as homozygous wild-type (WT/WT, healthy), heterozygous (mut/WT, affected), or homozygous mutant (mut/mut, affected).

### 2.3. Statistical Analyses

Statistical analyses were performed on the two breeds separately using R software version 4.3.1 (R Core Team, Vienna, Austria). To assess differences in genotypic frequencies among groups, the Chi-square test was applied using the function “chisq.test” in R (R Core Team). Statistical significance was set at *p* < 0.05. For statistically significant differences, post hoc pairwise comparisons were performed using Fisher’s exact test for each pair of groups. To account for multiple comparisons, *p*-values were adjusted using the Bonferroni correction [20]. A significance threshold of *p* < 0.05 was used to determine statistical significance.

Moreover, the allelic frequencies of the wild-type (WT) and mutant (mut) alleles were calculated separately for both breads using the following formulas:

$$f\left(allele\right)=\frac{2Nhomozygotes+NWTheterozygotes}{2N}$$

## 3. Results

### 3.1. Temporal Trend

**Figure 1:**
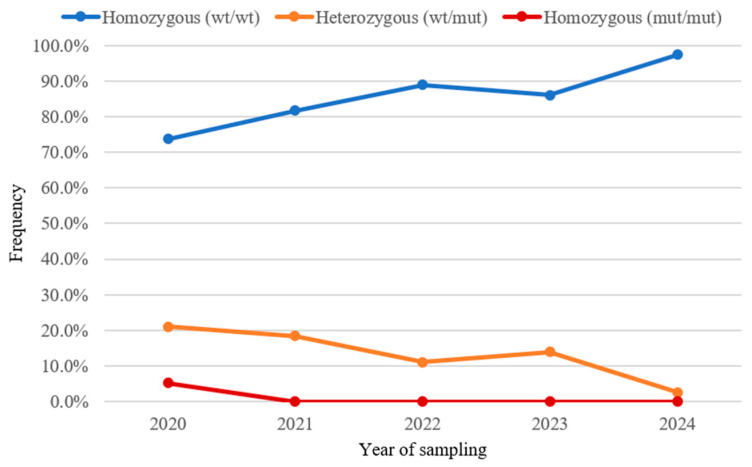
Temporal trend of genotypic frequencies (y-axis) of tested dogs from 2020 to 2024 (x-axis). The graph illustrates the genotypic frequency of homozygous healthy individuals (WT/WT) in blue, heterozygous individuals (WT/mut) in orange, and homozygous affected individuals (mut/mut) in red.

The results of this study show that, among the 233 Australian Shepherd dogs analyzed, the number of tested individuals increased significantly over the five-year period, rising from 19 tests in 2020 to 77 in 2024 (Table 1). Over time, there was a progressive increase in the proportion of healthy individuals (WT/WT), while the percentage of affected dogs—both heterozygous (WT/mut) and homozygous (mut/mut)—declined substantially (Figure 1). Specifically, the percentage of healthy dogs rose from 73.6% in 2020 to 97.4% in 2024, while the proportion of affected individuals decreased from 26.4% to 2.6%. Notably, the homozygous mutant genotype (mut/mut) was absent from the population after 2020.

Statistical analysis confirmed a significant difference in genotype distribution across the years (*p* = 0.0048). Post hoc analysis identified that this significance was primarily driven by differences between the earlier years and the final year of the study (2020 vs. 2024 and 2021 vs. 2024; adjusted *p*-values = 0.03 for both comparisons, Table 2).

Considering the cumulative genotypic distribution of all tested individuals across the study period, 88.4% were classified as healthy (WT/WT), 11.2% were heterozygous carriers (WT/mut), and only 0.4% were homozygous affected (mut/mut). Furthermore, the allelic frequency analysis revealed that the WT allele frequency was 93.99%, while the mut allele frequency was only 6.01%.

### 3.2. Sex

Among the 233 analyzed samples, 146 were from female dogs (63%), while 87 were from male dogs (37%). As reported in Table 3, the results indicate that in both sexes, the most represented group consists of healthy dogs (87% in females and 90.8% in males). Conversely, the total number of affected individuals accounted for 13% of females, of whom only one dog was homozygous, and 9.2% of males, with no homozygous cases.

The frequencies of affected and healthy dogs in the two sexes are comparable. Indeed, statistical analysis confirmed that differences in genetic distribution between sexes were not statistically significant (*p*-value = 0.94).

### 3.3. Coat Color

The association between hereditary cataracts and coat color was further explored. As shown in Table 4, the most common coat colors among the subjects in this study were blue merle (n = 78), black tricolor (n = 67), red merle (n = 30), and red tricolor (n = 35).

Analyzing the results, the frequency of individuals carrying the mutation responsible for hereditary cataracts is highest in black tricolor (14.9% heterozygosity and 1.5% homozygosity), blue merle (10.3%), red tricolor (17.1%), and tricolor undefined (20%). Nevertheless, statistical analysis revealed no significant association between coat color and genotypic frequency (*p*-value = 0.38).

### 3.4. Age

Most dogs were tested between 0 and 4 years old, with a peak in the 1–2 years of age range. Conversely, only a small percentage of dogs from older age groups, particularly those over 6 years old, were tested. The higher prevalence of individuals carrying at least one diseased allele in the 1–2 years of age group (Table 5) reflects the larger number of tests conducted in this age range. The observed frequency of the mutation across age groups is not statistically different (*p* value= 0.70), indicating that age does not influence genotype distribution, but rather reflects testing patterns and breeding-related decisions.

### 3.5. Miniature American Shepherds

Data on Miniature American Shepherds is available only from 2022 onwards, as no genetic tests for *HSF4* mutation were conducted on this breed in Italy during 2020 and 2021. The number of tested individuals per year was six in 2022, five in 2023, and two in 2024, totaling 13 dogs. All tested individuals were between one and three years old and were homozygous wild-type (WT/WT), indicating that none carried the *HSF4* mutation. Due to the small sample size, these results are purely descriptive and should be interpreted with caution. Based on this limited dataset, the estimated allelic frequency is 100% for the WT allele and 0% for the mutant allele, but no meaningful conclusion can be drawn regarding the true prevalence in the breed.

## 4. Discussion

The results of this study show a significant reduction in the frequency of the *HSF4* mutation associated with hereditary cataract in improvement in Australian Shepherds in Italy. Over the five-year study period, there was a steady increase in the number of tested dogs was observed, with a marked rise in the percentage of homozygous wild-type (WT/WT) individuals, reaching 97.4% in 2024. Conversely, the frequency of affected dogs, particularly homozygous mutant (mut/mut), steadily declined, with no cases observed after 2020. Statistical analysis confirmed the significance of this temporal trend, specifically highlighting differences between the earliest study years (2020 and 2021) and the most recent year (2024) (Table 2).

A similar trend was reported by Majchrákova et al. [21], who analyzed data from five European countries from 2012 to 2022, further supporting the hypothesis that genetic testing and selective breeding practices contribute to a reduction in the prevalence of the mutation. In detail, they did not find any mutant homozygotes and observed a decrease in the proportion of affected heterozygotes from approximately 35% in 2012 to 15% in 2022. This highlights the importance of early genetic screening in breeding programs.

The frequency of the causal mutation observed in the above-mentioned study aligns with previous research conducted in different dog populations. Mellersh et al. [11] analyzed 392 Australian Shepherds across 12 countries (Australia, Belgium, Canada, Czech Republic, Denmark, Finland, Germany, The Netherlands, New Zealand, Sweden, UK, and USA), reporting an overall homozygous wild-type frequency of 70.6%, 25.5% of heterozygous (WT/mut), and 3.8% of homozygous affected (mut/mut). Compared to these findings, the proportion of heterozygous dogs in our study is notably lower (11.2% vs. 25.5%), and the complete absence of homozygous affected individuals after 2020 represents a significant difference. This discrepancy could be attributed to differences in sample composition, as Mellersh et al. [11] included 99 (out of 392 dogs) with clinically confirmed cataracts, whereas our study focused on genotypic screening of the general population rather than clinically diagnosed cases.

In a smaller-scale study, Beckers et al. [22] analyzed 32 Australian Shepherd dogs in Belgium, reporting a mutant allele frequency of 7.8%, which is closer to our findings (6.1% mutant allele frequency). Their results also emphasized that hereditary cataracts remains one of the most frequent genetic disorders in the breed, second only to the MDR1 mutation.

Moreover, Majcháková et al. [21] examined a European population of 1641 Australian Shepherds from five different countries, reporting 76.72% WT/WT, 23.28% WT/mut, and 0% mut/mut, with an *HSF4* mutant allele frequency of 11.64%, which is slightly higher than the 6.1% observed in our study. This difference may reflect regional variations in breeding practices and genetic selection pressure. In fact, they found substantial differences in allele frequencies among European countries, with the highest frequency in the Czech Republic (17.6%) and the lowest in Germany (3.46%), suggesting potential differences among countries in breeding regulations or selection criteria. Interestingly, their study also confirmed that homozygous affected individuals (mut/mut) were absent across all countries analyzed (0%), aligning with our findings (0.4%).

Overall, these comparisons suggest that the *HSF4* mutation frequency is gradually decreasing in certain populations, likely due to increased awareness, genetic testing, and selective breeding efforts. However, regional differences persist, reinforcing the importance of continued genetic screening and responsible breeding practices to further reduce the prevalence of hereditary cataracts in the breed.

Regarding sex distribution, no significant differences were observed between males and females in terms of genetic profile. The percentage of healthy homozygous individuals (WT/WT) was comparable between sexes and the proportion of affected individuals did not indicate any clear sex-based predisposition. This is consistent with the fact that *HSF4* is an autosomal gene, meaning it is not sex-linked and therefore does not show sex-related inheritance patterns [11].

The relationship between coat color and mutation prevalence was also examined. The mutation appeared more frequently in black tricolor, blue merle, and red tricolor individuals, which are also the most represented coat colors in the study. Interestingly, no affected individuals were found among red merle dogs, despite their relatively high sample size. While this might suggest a lower association between red merle coat color and the mutation, a larger dataset would be needed to confirm this hypothesis. Overall, no statistically significant association was found between coat color and mutation status, reinforcing the need for further studies with a more balanced sample distribution.

When analyzing age distribution, most tested dogs were between 0 and 4 years old, with a peak in the 1–2 years range. This trend highlights breeders’ growing use of genetic testing before breeding, ensuring the early identification of affected individuals. In contrast, dogs over 6 years old were rarely tested, which may be due to a lack of perceived necessity for genetic screening in older dogs. Since the age of onset for hereditary cataracts varies, genetic testing remains the only reliable method to identify affected dogs before symptoms develop.

Similar to Australian Shepherds, the tested Miniature American Shepherds were all young dogs, with most data coming from the last three years. Notably, no official genetic test results were available in Italy before 2022, highlighting a recent increase in breeder awareness and testing practices for this breed. Although the mutation was not observed in any of the 13 tested individuals, the sample size is too small to draw any conclusions about its true frequency in the breed. Further monitoring and larger-scale genetic testing are necessary to determine whether this trend is representative of the broader population.

## 5. Conclusions

Overall, the findings of this study confirm a declining prevalence of the *HSF4* mutation in Australian Shepherd dogs in Italy, likely due to increased genetic testing and selective breeding efforts. The observed 6% mutant allele frequency is lower than previous European reports, suggesting that breeders are making informed decisions to reduce HC risk. However, the presence of 11.6% affected dogs indicates that continued vigilance is necessary to prevent the mutation from persisting in the breeding population. These results highlight the critical role of genetic testing in minimizing hereditary cataracts, reinforcing the importance of educating breeders on the impact of genetic selection on disease prevalence.

## Figures and Tables

**Table 1:** Annual distribution and frequencies of *HSF4* genotypes. Number (n) and frequencies (freq) of homozygous healthy individuals (WT/WT), heterozygous affected (WT/mut), and homozygous affected (mut/mut) dogs over the study period (2020–2024). The table also includes cumulative frequencies for the entire dataset. N of samples indicates the total number of dogs analyzed for each year of sampling.

Year of Sampling	N of Samples	Homozygous (WT/WT)		Heterozygous (WT/mut)		Homozygous (mut/mut)	
		n	freq	n	freq	n	freq
2020	19	14	73.6%	4	21.1%	1	5.3%
2021	49	40	81.6%	9	18.4%	0	0.0%
2022	45	40	88.9%	5	11.1%	0	0.0%
2023	43	37	86.0%	6	14.0%	0	0.0%
2024	77	75	97.4%	2	2.6%	0	0.0%
total	233	206	88.4%	26	11.2%	1	0.4%

**Table 2:** Post hoc comparisons of genotype frequencies across years. Post hoc pairwise comparisons between study years using Fisher’s Exact Test. Significant differences after Bonferroni correction (Adjusted *p*-value < 0.05) are marked *.

Comparison	*p*-Value	Adjusted *p*-Value
2020 vs. 2021	0.33	1
2020 vs. 2022	0.16	1
2020 vs. 2023	0.18	1
2020 vs. 2024	0.003	0.03 *
2021 vs. 2022	0.39	1
2021 vs. 2023	0.78	1
2021 vs. 2024	0.003	0.03 *
2022 vs. 2023	0.75	1
2022 vs. 2024	0.09	0.99
2023 vs. 2024	0.02	0.24

**Table 3:** *HSF4* genotype frequencies by sex. Number (n) and frequencies (freq) of homozygous healthy individuals (WT/WT), heterozygous affected (WT/mut), and homozygous affected (mut/mut) dogs by sex. N of samples indicates the total number of dogs analyzed for each sex.

Sex	N of Samples	Homozygous (WT/WT)		Heterozygous (WT/mut)		Homozygous (mut/mut)	
		n	freq	n	freq	n	freq
Female	146	127	87.0%	18	12.3%	1	0.7%
Male	87	79	90.8%	8	9.2%	0	0.0%

**Table 4:** *HSF4* genotype frequencies by coat color. Number (n) and frequencies (freq) of homozygous healthy individuals (WT/WT), heterozygous affected (WT/mut), and homozygous affected (mut/mut) dogs by the coat color. N of samples indicates the total number of dogs analyzed for each coat color.

Coat Color	N of Samples	Homozygous (WT/WT)		Heterozygous (WT/mut)		Homozygous (mut/mut)	
		n	freq	n	freq	n	freq
Black and white	2	2	100.0%	0	0.0%	0	0.0%
Black tricolor	67	56	83.6%	10	14.9%	1	1.5%
Blue merle	78	70	89.7%	8	10.3%	0	0.0%
Black and tan	10	10	100.0%	0	0.0%	0	0.0%
Red	2	2	100.0%	0	0.0%	0	0.0%
Red merle	30	30	100.0%	0	0.0%	0	0.0%
Red tricolor	35	29	82.9%	6	17.1%	0	0.0%
Red and tan	3	3	100.0%	0	0.0%	0	0.0%
Tricolor (undefined)	5	4	80.0%	1	20.0%	0	0.0%
Undetermined	1	0	0.0%	1	100.0%	0	0.0%

**Table 5:** *HSF4* genotype frequencies by age. Number (n) and frequencies (freq) of homozygous healthy individuals (WT/WT), heterozygous affected (WT/mut), and homozygous affected (mut/mut) dogs by the age.

Age	N of Samples	Homozygous (WT/WT)		Heterozygous (WT/mut)		Homozygous (mut/mut)	
		n	freq	n	freq	n	freq
<1 year	22	20	90.9%	2	9.1%	0	0.0%
1 year	67	57	85.1%	10	14.9%	0	0.0%
2 years	72	64	88.9%	8	11.1%	0	0.0%
3 years	40	37	92.5%	3	7.5%	0	0.0%
4 years	18	15	83.3%	2	11.1%	1	5.6%
5 years	8	7	87.5%	1	12.5%	0	0.0%
6 years	3	3	100.0%	0	0.0%	0	0.0%
7 years	1	1	100.0%	0	0.0%	0	0.0%
8 years	1	1	100.0%	0	0.0%	0	0.0%
12 years	1	1	100.0%	0	0.0%	0	0.0%

## Data Availability

Data presented in the manuscript is available upon request to the authors.
